# Sputum Metabolites Associated with Nontuberculous Mycobacterial Infection in Cystic Fibrosis

**DOI:** 10.1128/msphere.00104-22

**Published:** 2022-04-28

**Authors:** Paul Breen, Madsen Zimbric, Kristopher Opron, Lindsay J. Caverly

**Affiliations:** a Department of Pediatrics, University of Michigan Medical School, Ann Arbor, Michigan, USA; b Department of Internal Medicine, University of Michigan Medical School, Ann Arbor, Michigan, USA; Albert Einstein College of Medicine

**Keywords:** nontuberculous mycobacteria, cystic fibrosis, metabolomics, microbiome

## Abstract

Nontuberculous mycobacterial (NTM) pulmonary infections in people with cystic fibrosis (CF) are associated with significant morbidity and mortality and are increasing in prevalence. Host risk factors for NTM infection in CF are largely unknown. We hypothesize that the airway microbiota represents a host risk factor for NTM infection. In this study, 69 sputum samples were collected from 59 people with CF; 42 samples from 32 subjects with NTM infection (14 samples collected before incident NTM infection and 28 samples collected following incident NTM infection) were compared to 27 samples from 27 subjects without NTM infection. Sputum samples were analyzed with 16S rRNA gene sequencing and metabolomics. A supervised classification and correlation analysis framework (sparse partial least-squares discriminant analysis [sPLS-DA]) was used to identify correlations between the microbial and metabolomic profiles of the NTM cases compared to the NTM-negative controls. Several metabolites significantly differed in the NTM cases compared to controls, including decreased levels of tryptophan-associated and branched-chain amino acid metabolites, while compounds involved in phospholipid metabolism displayed increased levels. When the metabolome and microbiome data were integrated by sPLS-DA, the models and component ordinations showed separation between the NTM and control samples. While this study could not determine if the observed differences in sputum metabolites between the cohorts reflect metabolic changes that occurred as a result of the NTM infection or metabolic features that contributed to NTM acquisition, it is hypothesis generating for future work to investigate host and bacterial community factors that may contribute to NTM infection risk in CF.

**IMPORTANCE** Host risk factors for nontuberculous mycobacterial (NTM) infection in people with cystic fibrosis (CF) are largely unclear. The goal of this study was to help identify potential host and bacterial community risk factors for NTM infection in people with CF, using microbiome and metabolome data from CF sputum samples. The data obtained in this study identified several metabolic profile differences in sputum associated with NTM infection in CF, including 2-methylcitrate/homocitrate and selected ceramides. These findings represent potential risk factors and therapeutic targets for preventing and/or treating NTM infections in people with CF.

## INTRODUCTION

Cystic fibrosis (CF) is an autosomal recessive disease caused by mutations in the cystic fibrosis transmembrane conductance regulator (CFTR) gene and is characterized by impaired mucociliary clearance, recurrent respiratory infections, and progressive lung function decline, leading to early mortality (1, 2). Currently, nontuberculous mycobacterial (NTM) infections affect about 20% of individuals with CF, with some studies estimating that rate to be as high as 32.7% (3). The prevalence rates of NTM infection in CF are increasing worldwide by as much as 5% per year (4–7). While CFTR dysfunction and structural lung disease (e.g., bronchiectasis) are known risk factors for NTM infection, limited information is available on risk factors associated with NTM infections among people with CF (8). Epidemiologic studies revealed some general trends in host NTM risk factors in CF, including higher lung function, a lower body mass index, and older age (5, 6, 9–12). Despite identification of these trends, current attempts to use patient-specific clinical data to determine NTM infection risk have been largely inconclusive (13, 14).

Exposure to NTM arises from environmental sources, including soil and surfaces exposed to water, such as tap water (15, 16). In ∼40% of patients (17), NTM infections (i.e., one or more airway cultures positive for NTM) result in a diagnosis of NTM pulmonary disease (i.e., signs and symptoms of clinical decline attributed to the NTM infection) (18, 19), which is further associated with significant morbidity, health care-associated costs, and burdens of care (20–22). In most cases, the NTM responsible for airway infection in CF are either Mycobacterium abscessus complex or Mycobacterium avium complex, while other species, such as Mycobacterium simiae, Mycobacterium kansasii, and Mycobacterium fortuitum, are isolated in CF less frequently (5, 23, 24). While the increasing use of CFTR modulators has helped treat the underlying cause of CF and increased life expectancy, CFTR modulators have not yet shown sustained reductions in prevalence rates of CF pathogen infections, including NTM (25–29). Continued elucidation of the underlying determinants of NTM infection in people with CF therefore remains a priority.

Recent studies in CF and non-CF bronchiectasis have identified associations between airway microbiota, NTM infection, and NTM pulmonary disease (30, 31). The goal of this study was to determine features of the airway microbiome and metabolome associated with NTM infection in CF. We hypothesized that airway microbiome and metabolome differ between people with CF with and without NTM infection and that these differences may represent host NTM infection risk factors.

## RESULTS

### Clinical data.

A total of 69 sputum samples were collected from 59 subjects; 42 samples were from 32 subjects belonged to the NTM case cohort (14 samples collected before incident NTM infection [pre-NTM] and 28 samples collected following incident NTM infection [post-NTM]), while 27 were from NTM-negative controls. A total of 10 subjects who were part of the NTM case group contributed two samples each, nine subjects contributed one pre- and one post-NTM sample each, and one subject provided two post-NTM samples. The 27 control subjects contributed one sample each.

Subject demographics and characteristics are described in Table 1 (data based on sample-specific comparisons except where indicated). The NTM cases and NTM-negative controls did not significantly differ in the majority of clinical characteristics, though the NTM cases tended to be younger with better lung function (median age, 25.9 years, ppFEV_1_ [percent predicted forced expiratory volume in 1 s], 63) compared to the controls (median age, 31.6 years; ppFEV_1_, 46) (age, *P* = 0.41; ppFEV_1_, *P* = 0.13). Rates of infection with other CF pathogens were similar between the groups. The majority of the NTM cases (∼60%) had M. avium complex infection; M. abscessus complex was the second most common (Table 2). The minority of NTM cases were diagnosed with NTM pulmonary disease (14.3% and 21.4% of the subjects in the pre-NTM and post-NTM groups, respectively), and only three of the subjects were on treatment for NTM infection at the time of sample collection. While the majority of clinical variables also did not differ between the clinical cohorts in the subset of 41 subjects that had 16S rRNA gene sequencing in addition to metabolomics performed on the samples, the NTM subjects were younger (20.68 versus 30.85 years) and had greater disease aggressiveness than the controls (age, *P* < 0.0001; disease aggressiveness, *P* = 0.018) (Table S1).

**TABLE 1:** Subject demographics and clinical characteristics

Characteristic	No. (%)^*a*^ for:		*P* value
	NTM-positive subjects (42 samples from 32 subjects)	NTM-negative controls (27 samples from 27 subjects)	
Age, yrs [median (IQR)]^*b*^	25.9 (20.2–37.8)	31.6 (23.5–39.3)	0.41
Sex (% male)^*c*^	40.6	59.3	0.19
CF genotype^*c*^			
F508del homozygous	16 (50.0)	11 (40.7)	0.40
F508del heterozygous	13 (40.6)	11 (40.7)	
Other	3 (9.4)	5 (18.5)	
ppFEV_1_ [median (IQR)]^*b*^	63 (43–75)	46 (36–66)	0.13
Disease aggressiveness			
Mild	18 (42.9)	17 (63.0)	0.22
Moderate	15 (35.7)	5 (18.5)	
Severe	9 (21.4)	5 (18.5)	
Acceptable BMI^*b*^	25 (59.5)	15 (55.6)	0.81
Current CF respiratory cultures^*b*^			
P. aeruginosa	17 (40.5)	15 (55.6)	0.32
MRSA	11 (26.2)	4 (14.8)	0.37
MSSA	18 (49.2)	12 (44.4)	1
S. maltophilia	9 (21.4)	3 (11.1)	0.34
* Achromobacter* spp.	2 (4.8)	1 (3.7)	1
* Burkholderia* spp.	0 (0)	3 (11.1)	0.06
Aspergillus spp.	10 (23.8)	6 (22.2)	1
CF respiratory cultures, ≥1 positive^*d*^			
P. aeruginosa	26 (61.9)	20 (74.1)	0.43
MRSA	16 (38.1)	7 (25.9)	0.43
MSSA	25 (59.5)	19 (70.4)	0.45
S. maltophilia	19 (45.2)	5 (18.5)	0.04^*e*^
* Achromobacter* spp.	5 (11.9)	4 (14.8)	0.73
* Burkholderia* spp.	1 (2.4)	3 (11.1)	0.29
Aspergillus spp.	20 (47.6)	14 (51.9)	0.81
Diagnosis of CF-related diabetes^*b*^	18 (42.9)	5 (18.5)	0.04^*e*^
Chronic azithromycin^*b*^	24 (57.1)	21 (77.8)	0.12
Chronic inhaled antibiotics^*b*^	21 (50.0)	20 (74.1)	0.08
Inhaled steroids^*b*^	33 (78.6)	16 (59.3)	0.11
CFTR modulators^*b*^	15 (35.7)	8 (29.6)	0.79
Clinical state^*b*^			
Baseline	18 (42.9)	13 (48.1)	0.48
Exacerbation	9 (21.4)	8 (29.6)	
Treatment	11 (26.1)	4 (14.8)	
Recovery	4 (9.5)	2 (7.4)	

a Except where noted otherwise. IQR, interquartile range.

b At sample collection.

c Patient-specific comparison.

d Within 2 years prior to sample collection.

e Statistically significant.

**TABLE 2:** NTM-related clinical data

Parameter	No. (%) in group	
	Pre-NTM (*n* = 14)	Post-NTM (*n* = 28)
Species^*a*^		
M. avium complex	9 (64.3)	16 (57.1)
M. abscessus complex	0 (0)	8 (28.6)
Other	5 (35.7)	5 (17.9)
Age (yrs) relative to initial positive NTM culture [median (IQR)]	−0.49 (−0.80 to −0.29)	0.81 (0.0083 to 2.21)
NTM pulmonary disease^*b*^	2 (14.3)	6 (21.4)
NTM therapy^*c*^	0 (0)	3 (10.7)

a Percentages may not total 100% due to mixed-species infections.

b Initiated antimycobacterial therapy within 2 years of sample collection (post-NTM group) or 2 years of first positive NTM culture (pre-NTM group).

c At time of sample collection.

10.1128/msphere.00104-22.2 TABLE S1 Demographics and clinical characteristics for subjects with microbiome and metabolome data. *, statistically significant; 1, at sample collection; 2, within 1 year prior to sample collection; 3, within 2 years prior to sample collection; 4, at least 4 weeks of treatment within 1 year prior to sample collection. Download Table S1, DOCX file, 0.02 MB. Copyright © 2022 Breen et al. 2022 Breen et al. https://creativecommons.org/licenses/by/4.0/ This content is distributed under the terms of the Creative Commons Attribution 4.0 International license.

### Metabolomics.

**FIG 1:**
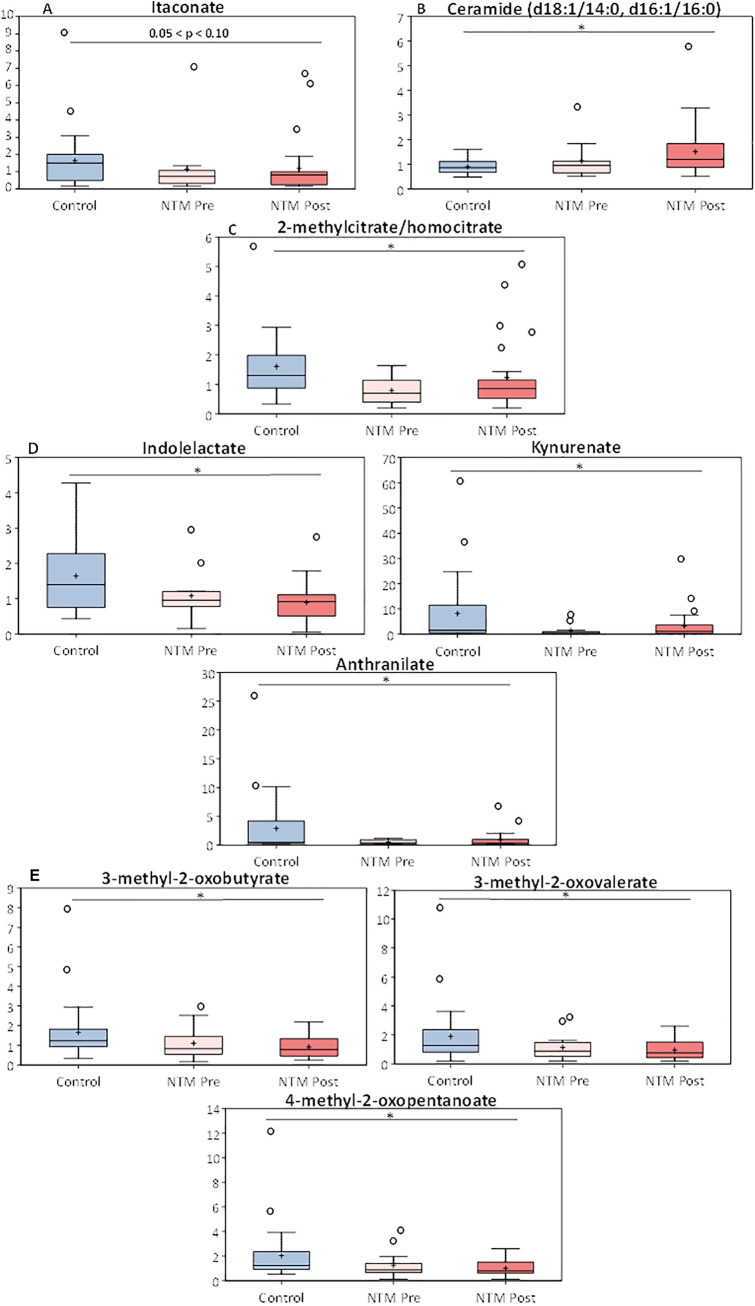
Metabolic profile of itaconate and various ceramides before and after NTM infection in CF patients and in NTM-negative CF controls. Box plots displaying the scaled intensities of (A) itaconate, (B) ceramides, (C) 2-methylcitrate/homocitrate, (D) tryptophan metabolites, and (E) branched-chain amino acid metabolites between subjects without NTM (Ctrl) and subjects with NTM infection (NTM Pre and Post). The box plots display the minimum and maximum distribution, the limits of the upper and lower quartile, the median, mean (+) and the extreme data points (○). Data were normalized to sample mass extracted, log transformed, and compared between subjects with and without NTM using Welch’s two-sample *t* tests and adjustment for multiple comparisons. *, *P* < 0.05 and *q* < 0.2.

The complete metabolic data set for all 902 biochemicals detected is available at https://github.com/caverlyl/NTM_metabolomics. Multiple metabolites significantly differed (i.e., *P* < 0.05 and *q* < 0.2) between the NTM case and control cohorts (Table 3). The NTM case cohort had trends toward lower levels of itaconate (an anti-inflammatory metabolite involved in macrophage activation) (*P* = 0.07; *q* = 0.228), significantly higher levels of C_18_ ceramides (sphingolipids involved in inflammation and cell signaling) (*P* < 0.05; *q* < 0.2), and significantly decreased levels of 2-methylcitrate/homocitrate (a metabolite of the methylcitrate pathway) (*P* = 0.002; *q* = 0.111) (Fig. 1A to C). Samples from the NTM case cohort also had decreases in certain tryptophan associated metabolites (Fig. 1D), decreased branched-chain amino acid metabolites (Fig. 1E), and increases in other compounds involved in phospholipid metabolism (*P* < 0.05; *q* ≤ 0.172 for all mentioned metabolites). Additionally, certain metabolites were also found to be significantly higher in both the pre- and post-NTM sputum samples than the controls. These metabolites include amino acids, such as serine and threonine; compounds involved in histidine, lysine, tryptophan, branched-chain, and aromatic amino acid metabolism; long-chain fatty acids and lipid metabolites; and pyrimidine metabolism compounds. Last, a number of dipeptide metabolites (e.g., leucylglycine, phenylalanylalanine, and valylglycine) were found to be significantly higher in the control group than the post-NTM group (*P* < 0.05; *q* < 0.2 for all listed metabolites). A complete list of all metabolites detected along with their statistical comparisons and values can be found in Table S2.

**TABLE 3:** Statistical summary of significantly altered biochemicals in sputum metabolites between subjects with and without NTM infection^*a*^

Test	Comparison	No. of biochemicals for which:			
		*P* ≤ 0.05		0.05 < *P* < 0.10	
		Total	No. increased; no. decreased	Total	No. increased; no. decreased
Welch’s two-sample *t* test	Pre-NTM vs. ctrl	58	7; 51	73	11; 62
	Post-NTM vs. ctrl	70	16; 54	79	17; 62
	All NTM vs. ctrl	138^*b*^	21; 117	67	17; 50
	Post-NTM vs. pre-NTM	14	8; 6	26	11; 15
Paired *t* test	Post-NTM vs. pre-NTM	26	17; 9	31	17; 14

a Biochemicals (811 compounds of known identity [named biochemicals] and 91 compounds of unknown structural identity [unnamed biochemicals]) that achieved statistical significance (*P* ≤ 0.05), as well as those approaching significance (0.05 < *P* < 0.10).

b This value falls within the 20% FDR (*q* < 0.2).

10.1128/msphere.00104-22.3 TABLE S2 Results of metabolomics analysis. Download Table S2, XLSX file, 0.3 MB. Copyright © 2022 Breen et al. 2022 Breen et al. https://creativecommons.org/licenses/by/4.0/ This content is distributed under the terms of the Creative Commons Attribution 4.0 International license.

### Microbiome and metabolomic data integration.

**FIG 2:**
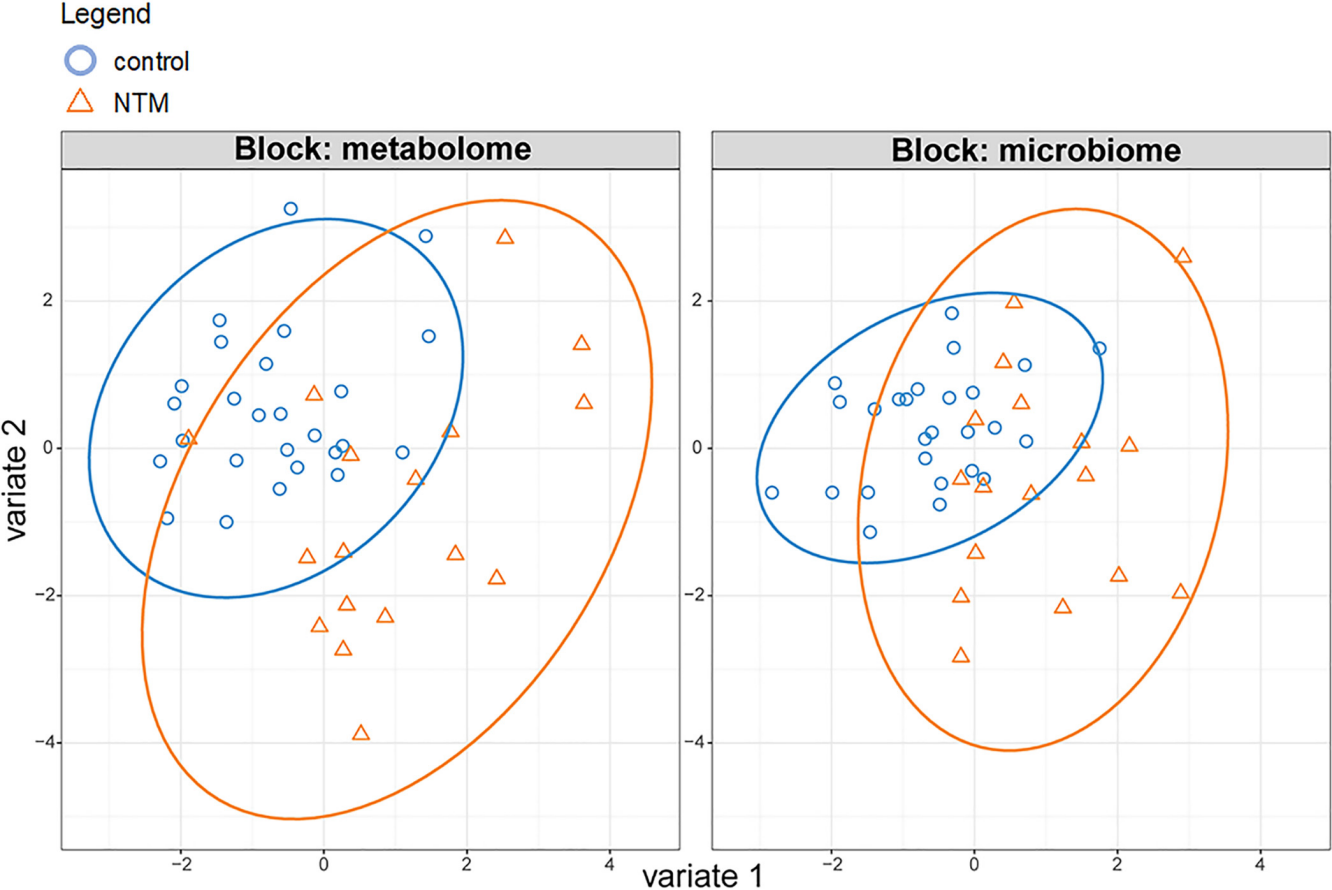
NTM cases display significantly different microbial and metabolomic profiles from NTM-negative controls. Separate sPLS-DA (DIABLO) ordinations for microbiome and metabolome (2 classes). Orange, NTM positive, blue, control.

We next sought to identify relationships between the sputum metabolites and the bacterial community profiles in the subset of samples (*n* = 43 samples and 41 subjects) that also had 16S rRNA gene sequencing. To identify the microbial and metabolomics profiles that were most discriminating between the NTM cases and controls, DIABLO was utilized to integrate the metabolomics and microbial data into one supervised analysis. The sparse partial least-squares discriminant analysis (sPLS-DA) ordinations show a clear separation between NTM case and control samples (Fig. 2). To support the sPLS-DA results, analysis of variance (ANOVA) with a Benjamini-Hochberg correction applied to the calculated *q* values was also used to test for differentially abundant features and identified multiple metabolites that differed between the NTM case and control groups, with lactobacillic acid displaying the smallest *P* value (*P* = 7.95E−05; *q* = 0.072) (Table S3). The separation of the NTM case and control groups by ordination is further supported by high accuracy (>90%) of classification on the sample set, with 21 of the 26 control samples being classified as controls and 12 of the 17 NTM samples being classified as NTM (Table S4). A supplemental PERMANOVA figure is also included to further support the sPLS-DA results (Fig. S1). This separation is borderline significant, with a *P* value of 0.052. Based on the classification of the sample set, the individual ordinations perform about as well as the combined ordination for classification (Fig. S2 and S3). Lastly, to further support the DIABLO results, generalized linear model (GLM) analysis with LASSO regularization was utilized to identify variables that can be classified as important predictors based on their coefficient value. As with the ANOVA, many of the identified features correspond to those detected utilizing the DIABLO analysis, specifically *Veillonella* (coefficient value = 0.367) (Table S5).

10.1128/msphere.00104-22.4 TABLE S3 Significant results of the 2-group ANOVA of metabolomics (115 samples). Download Table S3, DOCX file, 0.02 MB. Copyright © 2022 Breen et al. 2022 Breen et al. https://creativecommons.org/licenses/by/4.0/ This content is distributed under the terms of the Creative Commons Attribution 4.0 International license.

10.1128/msphere.00104-22.5 TABLE S4 Confusion matrix of results for consensus classification using microbial and metabolomic ordinations. Download Table S4, DOCX file, 0.02 MB. Copyright © 2022 Breen et al. 2022 Breen et al. https://creativecommons.org/licenses/by/4.0/ This content is distributed under the terms of the Creative Commons Attribution 4.0 International license.

10.1128/msphere.00104-22.6 TABLE S5 Cross-validated GLM with LASSO approach for feature selection using microbial and metabolic features of NTM case and control groups. Misclassification error = 0.23255. Only variables with nonzero coefficients are listed. Download Table S5, DOCX file, 0.02 MB. Copyright © 2022 Breen et al. 2022 Breen et al. https://creativecommons.org/licenses/by/4.0/ This content is distributed under the terms of the Creative Commons Attribution 4.0 International license.

10.1128/msphere.00104-22.7 FIG S1 PERMANOVA test of control versus NTM samples by ordination for both the microbiome and metabolome. PCoA with Euclidean distances with all potential microbial and metabolomic features. Significant testing by PERMANOVA. Download FIG S1, TIF file, 2.6 MB. Copyright © 2022 Breen et al. 2022 Breen et al. https://creativecommons.org/licenses/by/4.0/ This content is distributed under the terms of the Creative Commons Attribution 4.0 International license.

10.1128/msphere.00104-22.8 FIG S2 Separation of control versus NTM samples by ordination for both the microbiome and metabolome. ROC curves comparing control and NTM samples. (A) Microbiome, component 1; (B) microbiome, component 2; (C) metabolome, component 1; (D) metabolome, component 2. The outcome results are based on Kruskal-Wallis test results for significantly differentially abundant microbial and metabolomic features. Download FIG S2, TIF file, 0.5 MB. Copyright © 2022 Breen et al. 2022 Breen et al. https://creativecommons.org/licenses/by/4.0/ This content is distributed under the terms of the Creative Commons Attribution 4.0 International license.

10.1128/msphere.00104-22.9 FIG S3 Separation of the experimental groups by ordination for both the microbiome and metabolome. ROC curves comparing control, NTM, and other samples. (A) microbiome, component 1; (B) microbiome, component 2; (C) metabolome, component 1; (D) metabolome, component 2. Download FIG S3, TIF file, 0.5 MB. Copyright © 2022 Breen et al. 2022 Breen et al. https://creativecommons.org/licenses/by/4.0/ This content is distributed under the terms of the Creative Commons Attribution 4.0 International license.

**FIG 3:**
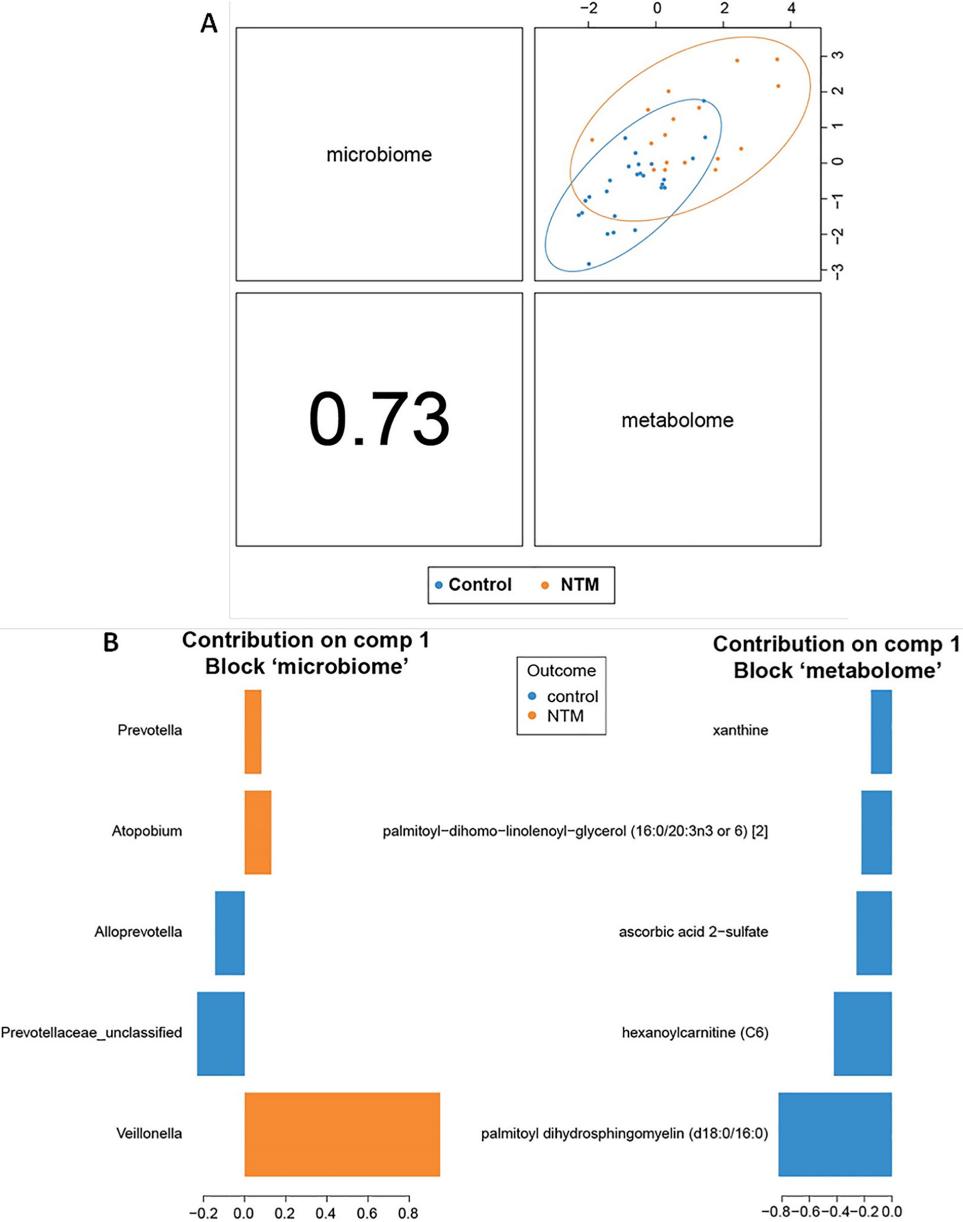
NTM infections in CF patients display significantly different microbial and metabolomic profiles from control samples when examining combined ordinations of component 1. (A) Global overview of the correlation structure of the combined sPLS-DA (DIABLO) ordination components of the microbiome and metabolome. (Left) Visual representation of split between groups by microbiome (*y* axis) and metabolome (*x* axis) for component 1. Correlation between microbial and metabolomic aspects of component 1 is shown in the bottom left panel. (Right) Loading weights indicating relative contribution of individual features to component 2. Colors indicate the class of the sample with the maximum observed value for each feature. (B) Summary of component 1 of sPLS-DA (DIABLO) ordination.

**FIG 4:**
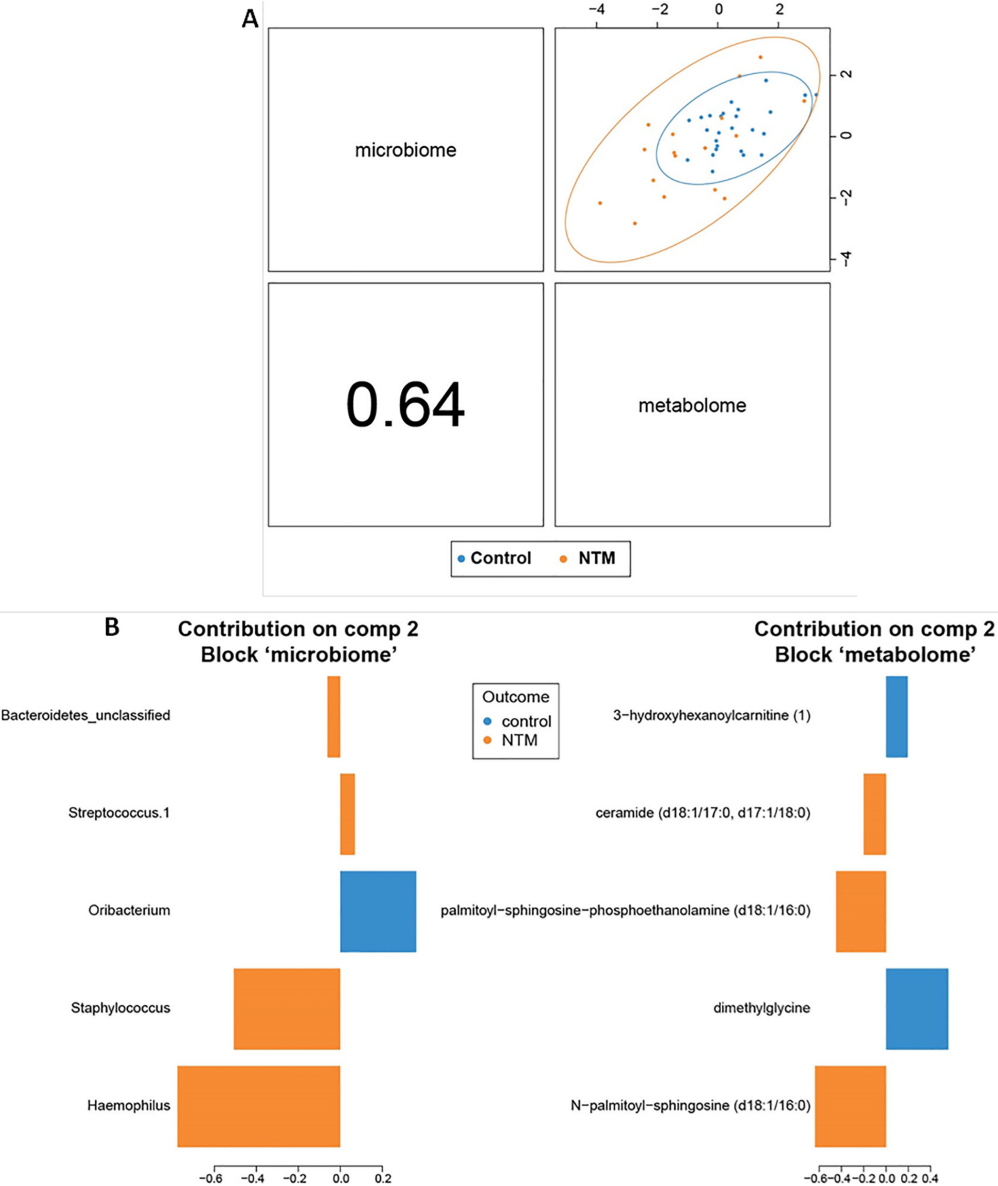
NTM infections in CF patients display significantly different microbial and metabolomic profiles from control samples when examining combined ordinations of component 2. (A) Global overview of the correlation structure of the combined sPLS-DA (DIABLO) ordination components of the microbiome and metabolome. (Left) Visual representation of split between groups by microbiome (*y* axis) and metabolome (*x* axis) for component 2. Correlation between microbial and metabolomic aspects of component 2 is shown in the bottom left panel. (Right) Loading weights indicating relative contribution of individual features to component 2. Colors indicate the class of the sample with the maximum observed value for each feature. (B) Summary of component 2 of sPLS-DA (DIABLO) ordination.

**FIG 5:**
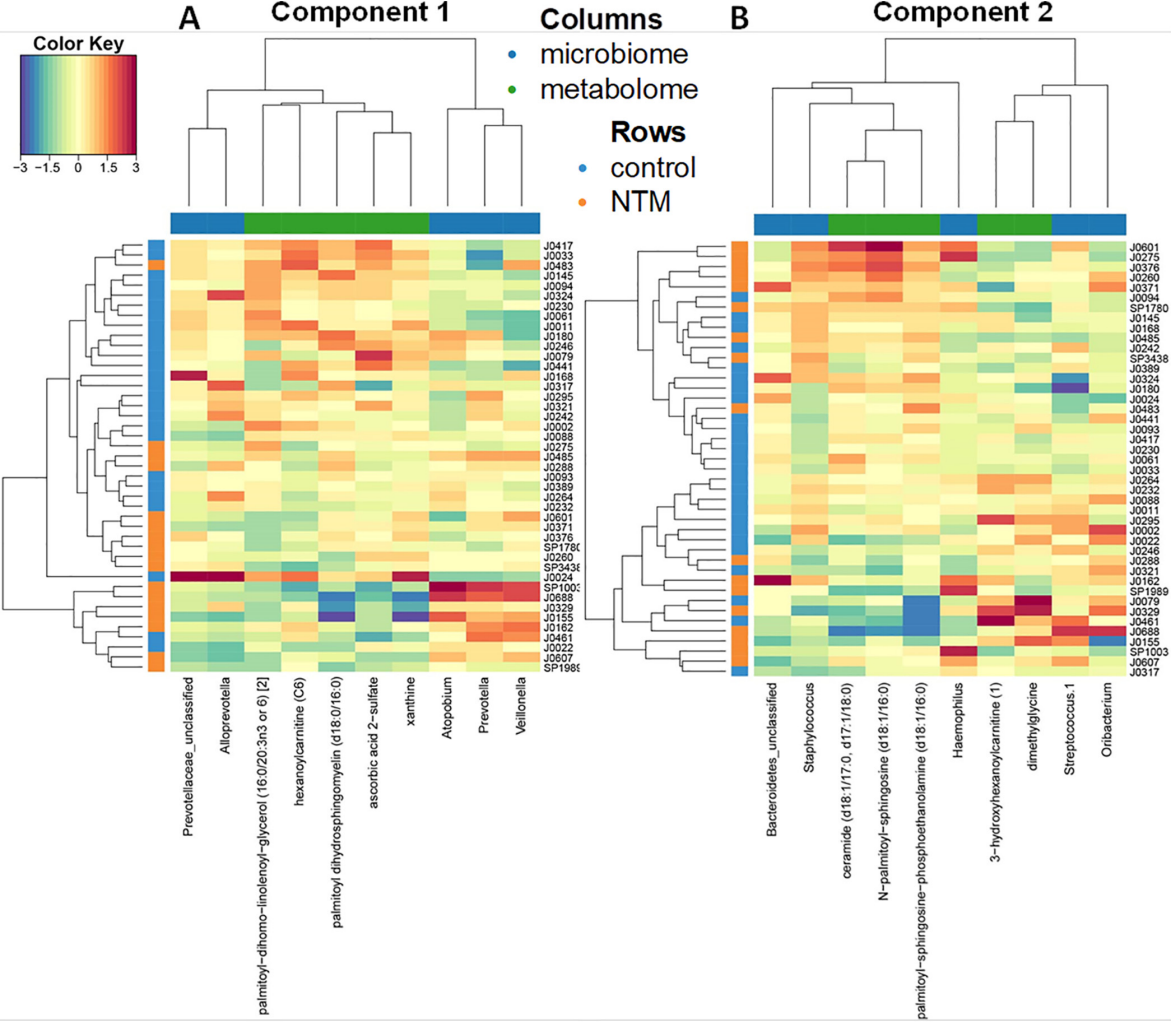
Heat maps displaying the metabolomics and 16S microbiome features. Hierarchical clustering with Euclidean distances was applied to rows and columns, as indicated by the dendrogram on the top and left sides. Values for each feature are standardized to zero mean and unit variance. Features listed for component 1 (A) and 2 (B) of sPLS-DA (DIABLO).

To further identify correlations between the microbial and metabolic features that differed between the NTM case and control groups, we next looked at the first two components of the sPLS-DA ordination. The two components of the sPLS-DA ordination are made up of the features that provide the greatest separation of the classes. Each component is made up of correlated microbial and metabolomic features (Fig. 3 and 4). For example, the components in Fig. 3 are based on the *x* axis values of the metabolome and microbiome in Fig. 2, while the components in Fig. 4 are constructed from the *y* axis values of the metabolome and microbiome in Fig. 2. For component 1 (Fig. 3), some of the microbial features are more tightly clustered with metabolic features; in the initial split of the hierarchical clustering of features, three operational taxonomic units (OTUs) (*Veillonella*, *Atopobium*, and *Prevotella*) formed a cluster, while the other two OTUs (*Prevotellaceae*_unclassified and *Alloprevotella*) formed a second cluster that includes all of the metabolites (Fig. 5A). For component 2 (Fig. 4), microbial and metabolic features are mixed fairly evenly across clusters (Fig. 5B). The five contributing features to the microbial portion of component 2 are Haemophilus, Staphylococcus, *Oribacterium*, Streptococcus.1, and *Bacteroidetes*_unclassified; *Oribacterium* is higher in the control group, while the other four are higher in the NTM group (Fig. 4B). Note that based on the coloring in Fig. 3B, the microbial features that increase with the component score are sometimes higher in control samples and other times higher in NTM samples (i.e., the colors of bars on the same side vary). For example, Streptococcus.1, is higher in the NTM group despite the fact that it trended in the same direction as *Oribacterium*, which is higher in the control group. This may indicate that some of these less impactful OTUs are not particularly good features for the classification of these samples. Instead, the value of this component for classification may come primarily from the first three features. The metabolic portion of component 2 (Fig. 4B) is primarily driven by *N*-palmitoyl-sphingosine (d18:1/16:0), dimethylglycine, palmitoyl-sphingosine-phosphoethanolamine (d18:1/16:0), ceramide (d18:1/17:0, d17:1/18:0), and 3-hydroxyhexanoylcarnitine (1). Dimethylglycine and 3-hydroxyhexanoylcarnitine (1) are more highly expressed in control samples, while the other three are more highly expressed in NTM samples (Fig. 4B).

**FIG 6:**
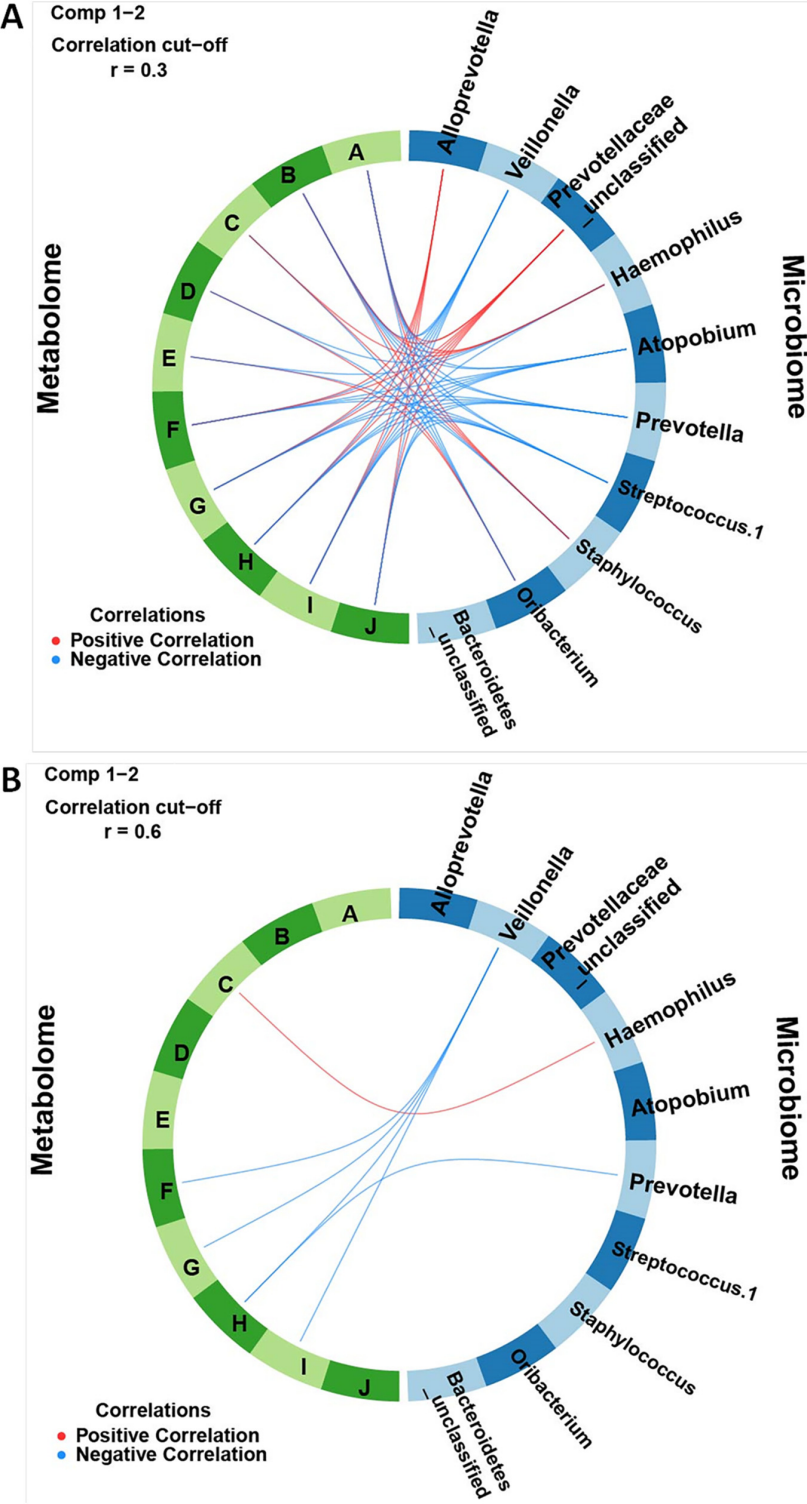
Circos plot of correlations between microbiome and metabolome. Positive and negative correlations of (A) 0.3 correlation cutoff and (B) 0.6 correlation cutoff, identified between microbial OTUs and metabolites of components 1 and 2 of sPLS-DA. Microbial OTUs are labeled by rank abundance and taxonomic classification. Labels: A, palmitoyl sphingosine-phosphoethanolamine (d18:1/16:0); B, ceramide (d18:1/17:0, d17:1/18:0); C, glycosyl-*N*-palmitoyl-sphingosine (d18:1/16:0); D, dimethylglycine; E, 3-hydroxyhexanoylcarnitine (1); F, xanthine; G, ascorbic acid 2-sulfate; H, palmitoyl dihydrosphingomyelin (d18:0/16:0); I, hexanoylcarnitine (C_6_); J, palmitoyl-dihomo-linolenoyl-glycerol (16:0/20:3n3 or 6).

Heat maps provide a detailed view of the features that make up each component and how they separate the classes (Fig. 5A and B). Certain OTUs and metabolites that contribute the most to the separation of classes are those that cluster near a feature from the other omics data set. For example, in component 1, *Prevotellaceae*_unclassified and *Alloprevotella* are closely clustered with metabolites based on their expression (Fig. 5A), and they all contribute significantly to component 1 (Fig. 3B and 5A). Haemophilus, *Prevotellaceae*_unclassified, Staphylococcus, and *Alloprevotella* are also generally positively correlated with many of the most discriminating metabolites (palmitoyl-sphingosine-phosphoethanolamine [d18:1/16:0], ceramide [d18:1/17:0, d17:1/18:0], and *N*-palmitoyl-sphingosine [d18:1/16:0]) (Fig. 6A and B). Similarly, the most discriminating feature for the metabolic component 1 (palmitoyl dihydrosphingomyelin [d18:0/16:0]) is the metabolite that most resembles OTUs based on expression (i.e., its contribution to component 1 is the largest of the listed metabolites) (Fig. 3B). The other most discriminating OTUs are those that are negatively correlated with and cluster furthest from the metabolites. These include, in order of decreasing contribution to component 1, *Veillonella*, *Atopobium*, and *Prevotella* (Fig. 3B). All of these OTUs (and Streptococcus.1) are negatively correlated with multiple metabolic features (Fig. 6A and B). The metabolic features in component 1 are all more highly expressed in the control group than the NTM group (Fig. 3B).

### Controls.

Sequencing error rates based on analyses of mock communities ranged from 0.000177 to 0.00412. No significant signal was detected from either the water blanks or reagent controls that would suggest PCR or reagent contamination of sputum samples (Fig. S4).

10.1128/msphere.00104-22.10 FIG S4 Error analysis and controls. Highest abundance OTUs in sequenced negative controls compared to mean abundance in experimental samples. (A) Reagent control RC022119; (B) reagent control RC081319; (C) water blank run C3 plate B; (D) water blank run C4 plate C. Error bars display standard errors of the means. OTUs are clustered separately for each control. Download FIG S4, TIF file, 0.5 MB. Copyright © 2022 Breen et al. 2022 Breen et al. https://creativecommons.org/licenses/by/4.0/ This content is distributed under the terms of the Creative Commons Attribution 4.0 International license.

## DISCUSSION

We identified metabolic patterns in sputum that significantly differ between people with CF with and without NTM infection. The untargeted metabolomics analysis revealed numerous metabolites that significantly differed between the NTM case and NTM negative-control subjects. These include metabolites that play important roles in the host immune response and in bacterial proliferation. Identification of associations between sputum metabolites and NTM infection, including metabolites in the NTM cohort prior to NTM infection onset that differed from NTM-negative controls, is hypothesis generating for future studies to address knowledge gaps in host risk factors for NTM infection in people with CF.

DIABLO with a supervised analysis was utilized to integrate the results from the untargeted metabolomics on the subset of the patients and samples with 16S microbial sequencing results. Analyzing the data using both an individual and combined omics approaches, our sPLS-DA analysis displayed a clear separation between NTM-case and control samples. In order to capture the full scope of features that separated the case and control groups, the correlated microbial and metabolomic features were categorized into two components which displayed significant correlations between selected bacterial OTUs and metabolites.

Metabolites that were found to significantly or nearly significantly differ from control and pre-NTM and post-NTM samples included itaconate, ceramides (C_18_ backbones), 2-methylcitrate/homocitrate, and intermediates in the utilization of both aromatic and branched-chain amino acid metabolism. The finding that itaconate, a compound produced by macrophages following activation by lipopolysaccharides (LPS) and/or interferons that also has anti-inflammatory properties, is lower in the NTM case group (both pre- and post-NTM infection) is especially interesting (32, 33). Studies have found that itaconate can inhibit Mycobacterium tuberculosis proliferation by inhibiting the glyoxylate shunt enzyme, isocitrate lyase (ICL) (34–36). However, other studies have shown that M. tuberculosis has the ability to either digest or dissimilate large quantities of itaconate into pyruvate and acetyl coenzyme A (acetyl-CoA) (33, 37, 38). Importantly, itaconate is able to inhibit the growth of M. tuberculosis when the bacterium is in minimal medium supplemented with short-chain fatty acids (SCFAs), compounds which are released by anaerobic bacteria in the CF airway (39). While data on the interactions of NTM and itaconate are limited and beyond the scope of this study, we hypothesize that a similar inhibitory effect may occur.

Selected ceramides were also increased NTM case group (both pre- and post-NTM infection) compared to the control group. Currently, data on interactions between NTM infections and the acid sphingomyelinase/ceramide system are lacking (40). One study by Utermöhlen et al. demonstrated that acid sphingomyelinase-deficient mice are more resistant to lethal infections with M. avium than wild-type mice (41). Ceramides in CF are generally important for the regulation of cytokines and inflammation, but as with itaconate, some conflicting evidence exists (42, 43). Some studies have found that ceramides accumulate in the CF airways, which in turn causes inflammation and an increased susceptibility to bacterial infections (42). Conversely, other studies have found that the increased presence of ceramides in CF patients helps to reduce overall inflammation and cytokine production in the lungs (44). In mice, for example, one study found that CF mice had elevated levels of peribronchial macrophages and neutrophils, along with increased concentrations of inflammatory markers such as interleukin 1 (IL-1) and the mouse IL-8 homolog, KC, in comparison with wild-type mice. Once the ceramide levels were normalized by genetic inhibition of acid sphingomyelinase (an enzyme responsible for catalyzing the breakdown of sphingomyelin to ceramide and phosphorylcholine), all of the aforementioned molecular inflammatory markers were normalized as well (44). While the reason for the rise in ceramide levels is unclear, studies have established that ceramide regulation in CF patients is dysregulated, and that multiple stimuli, such as UV irradiation, heat, cytokines, oxidative stress, and LPS, can generate ceramides (43).

The decrease in the metabolite 2-methylcitrate/homocitrate is also an intriguing result, as the methylcitrate pathway is the main pathway involved in propionate utilization (45). An increasing body of evidence has demonstrated that mycobacteria utilize fatty acids during infection (46, 47). Propionate, a SCFA, can serve as a nutrient and carbon source for many different species of bacteria; however, at high enough levels; the metabolite is toxic, highlighting the importance of the methylcitrate pathway (45–47). Moreover, the SCFA is a common by-product of bacterial fermentation, and with the observed increase in anaerobic bacteria preceding NTM infection, this suggests that propionate is an essential metabolite for NTM in the CF airway (45).

Observed decreases in aromatic and branched-chain amino acids (BCAAs) in the NTM case group are an expected finding, as both of these types of amino acids are essential for the growth of Mycobacterium (48, 49). Mycobacteria, like many other organisms, produce aromatic amino acids through the shikimate pathway, with the enzyme 3-deoxy-d-arabino-heptulosonate 7-phosphate synthase (DAH7PS) catalyzing the first step of the reaction (50, 51). However, mycobacteria possess only one isozyme of DAH7PS, which can be controlled by combinations of aromatic amino acids; this allows the DAH7PS to have a tunable response to changing metabolic demands when in the presence of all three aromatic amino acids (51). BCAA availability in the lung is extremely limited, and given the importance of these compounds in mycobacterial growth, it is not surprising that the levels drop even further in the presence of NTM species (49, 52).

In the analysis of component 1, all of the correlated metabolites that contributed to component 1 were significant only in the control group. However, certain OTUs significantly correlated with the NTM positive cohort; these OTUs include obligate and facultative anaerobic genera, such as *Veillonella*, *Atopobium*, and *Prevotella*, while an unclassified *Prevotellaceae* bacterium and *Alloprevotella* correlated with the control cohort. Additionally, an interesting clustering trend was observed when examining the heat maps associated with component 1: the two OTUs correlated with the control group (unclassified *Prevotellaceae* and *Alloprevotella*) both clustered with the metabolites that were identified as significant in this component analysis. While the reason for this is unclear, it is consistent with our prior research. Using a different cohort of subjects with CF and NTM infection, we identified an overall increasing relative abundance of *Veillonella*, *Prevotella*, and *Rothia* across longitudinal samples leading to incident NTM infection in subjects subsequently diagnosed with NTM pulmonary disease, compared to subjects without NTM pulmonary disease (31).

The results observed for component 1 suggest that the loss of significantly correlated metabolites could be a predisposing factor for NTM infection. For example, the inclusion of ascorbic acid 2-sulfate (a metabolite of vitamin C) in the control group is an interesting finding, as vitamin C has been shown to have antimicrobial properties against M. tuberculosis (53, 54). Additionally, inclusion of the metabolites hexanoylcarnitine and xanthine in the control group is also an intriguing finding, as carnitine compounds can be used as bacterial nutrients, while the purine compound xanthine has been shown to be a viable nitrogen source for M. smegmatis (55, 56). Other metabolites, however, such as palmitoyl-dihomo-linolenoyl-glycerol have less well defined roles. Whether the reduction of the aforementioned metabolites is leading to changes in the microbial community of the CF airway, thus creating a niche for NTM bacteria to proliferate or whether the changes in the microbial community lead to changes in the metabolome (or a mix of both) remains to be clarified.

For the component 2 analysis, bacterial OTUs such as Haemophilus, Staphylococcus, Streptococcus, and unclassified *Bacteroidetes* (the phylum to which *Prevotella* belongs) were all correlated with NTM infection, while *Oribacterium* was correlated with the control group. The findings for Haemophilus, Streptococcus, and *Bacteroidetes* match what was reported in our previous publication, while the correlation with Staphylococcus differs from it (31). A likely explanation for this discrepancy is that this analysis also included metabolites from the component analysis, which resulted in significantly different correlation patterns. Furthermore, the metabolites that are significantly correlated may contribute substantially to the growth of the bacterial OTUs in question. As with the component 1 analysis, a carnitine metabolite was again correlated with the control group along with dimethylglycine, a derivative of the amino acid glycine, and the bacterium *Oribacterium*, a member of the family *Lachnospiraceae*, which are some of main producers of short-chain fatty acids in the gut (57). The correlation of dimethylglycine with the control group is an interesting finding, as it very likely could serve as a nitrogen source for NTM, given the large amount of flexibility some mycobacteria (specifically M. tuberculosis) have in nitrogen utilization (58, 59). The findings from both component analyses, displaying distinct differences between the cases and controls that do not differ in bacterial composition but do in metabolic function and in microbial interactions, fit with the climax-attack model of CF infections (60). Future longitudinal studies will likely aid in elucidating mechanistic links between NTM, the CF airway microbial community, and the metabolome.

While our study did reveal significant differences between in sputum metabolites and microbiome between people with CF with and without NTM infection, we acknowledge study limitations. This was a relatively small, single-center, cross-sectional study which did not allow the establishment of temporal or cause-and-effect relationships. Samples had variability in the timing of sample collection in relation to NTM infection, and some subjects contributed multiple samples. While the NTM case and control groups overall did not significantly differ with regard to the majority of clinical variables known to be associated with airway microbiota, we acknowledge the possibility that any identified differences in the microbiota reflected unmeasured clinical variables, rather than NTM infection. Other important factors that may have contributed to the observed differences in the metabolomics profiles between post-NTM and control subjects could be the effects of NTM on the resident microbial community, NTM-directed therapeutics (though only three of the samples were collected during NTM treatment), and the aggressive antibiotics (not NTM directed) that are often given to people with CF with new NTM infections as part of the protocol to determine if NTM pulmonary disease (i.e., clinical decline attributed to the NTM infection, rather than other CF comorbidities) is present.

We acknowledge that the 20% false discovery rate (FDR) of our metabolomics analysis creates a possibility for false positives in some of the 138 metabolites found to be significantly different in the NTM versus control analysis, despite the *q* values meeting the FDR. The potential for false discovery can also be applied to the DIABLO analysis, despite the use of Benjamini-Hochberg-corrected *q* values. Additionally, while the ROC curves displayed a strong separation by components and our sPLS-DA approach produced high accuracy and classification of our data set, our sample size did not allow separate training and validation sets, and thus, these findings will need validation in independent cohorts. Future studies will take these limitations into consideration, allowing increased depth and power when analyzing the differences in the microbial communities of CF patients with and without NTM infection. Last, tests with training samples and external validation are needed to determine how much nonoverlapping information is contained in the two omics data sets.

While it is not clear at this time how these findings apply to other individuals with CF, or to NTM infections in other lung diseases, the data found in this study mirror what has been observed in previous studies, namely, that certain anaerobic bacteria are overrepresented in patients with NTM infection, and extends these observations to also include differences in sputum metabolites associated with NTM infection. Identified differences in these metabolites associated with NTM infection are hypothesis generating for their potential role in susceptibility to NTM infections and will be the subject of future studies. An increased understanding of the relationship and interactions between the airway microbiota and metabolome in the contexts of bacterial interactions and host response will be critical to continuing to advance understanding of the pathophysiology of NTM infections in individuals with CF, and ultimately to direct development of novel biomarkers and therapeutic approaches.

## MATERIALS AND METHODS

### Subjects, samples, and clinical data.

Sputum samples were collected from subjects diagnosed with CF and enrolled in a long-term, observational study of CF airway microbiota at Michigan Medicine with approval from the Institutional Review Board (first approved 22 August 2016). Subjects who had undergone organ transplantation were excluded. Spontaneously expectorated sputum samples were collected in sterile containers at routine clinic visits. Samples were placed immediately on ice, then aliquoted, and frozen at −80°C in a biorepository within 4 h.

Clinical data were obtained through Michigan Medicine electronic medical records. NTM subjects were defined as subjects who had one or more respiratory samples that were acid-fast bacillus (AFB) culture positive for an NTM species. Sputum samples collected both before (pre-NTM) and after (post-NTM) incident NTM infection were selected from the biorepository. NTM-negative controls were defined as subjects who had never had a positive AFB culture, had a negative AFB culture at the time of sample collection, and had at least one negative AFB culture following the time of the included NTM-negative sample. All eligible NTM cases and NTM-negative controls from a 3-year period (2016 to 2019) were selected from the biorepository.

Clinical data collected included sex, CFTR genotype, diagnosis of CF-related diabetes, age, percent predicted forced expiratory volume in 1 s (ppFEV_1_), medication history, and airway culture results. A positive culture at the time of sample collection and a history of a positive culture within 2 years prior to sample collection were recorded for the following organisms: Pseudomonas aeruginosa, Stenotrophomonas maltophilia, Achromobacter xylosoxidans, methicillin-resistant Staphylococcus aureus (MRSA), methicillin-susceptible S. aureus (MSSA), *Burkholderia* spp., and Aspergillus spp. Treatment at the time of sample collection was determined for the following pharmacological therapies: chronic azithromycin, inhaled antibiotics (e.g., tobramycin), CFTR modulators, and inhaled steroids.

Clinical state at the time of sample collection was categorized using the baseline (B), exacerbation (E), treatment (T), and recovery (R) states as previously described (61). Aggressiveness of CF disease was categorized as mild, moderate, or severe by comparing ppFEV1 history preceding sample collection to a previously published age and lung function-dependent algorithm (62). Body mass index (BMI) was categorized as indicating risk for pediatric patients if they were below the 50th percentile on the CDC 2- to 20-years-old charts for appropriate age and sex, for adult males if the BMI was less than 23 kg/m^2^, and for adult females if the BMI was less than 22 kg/m^2^ (63, 64).

### Metabolomics.

Sputum samples were shipped on dry ice for short-chain fatty acid (SCFA) measurements and untargeted metabolomics (ultrahigh-performance liquid chromatography–tandem mass spectroscopy) at Metabolon, following their standard procedures. Samples were maintained at −80°C until processed. For SCFA analysis, sputum samples were spiked with stable labeled internal standards, homogenized, subjected to protein precipitation with an organic solvent, and then analyzed by liquid chromatography-tandem mass spectrometry (LC-MS/MS) for eight short-chain fatty acids: acetic acid (C_2_), propionic acid (C_3_), isobutyric acid (C_4_), butyric acid (C_4_), 2-methyl-butyric acid (C_5_), isovaleric acid (C_5_), valeric acid (C_5_), and caproic acid (hexanoic acid; C_6_). For untargeted metabolite measurements, samples were prepared using the automated MicroLab STAR system from Hamilton Company. All methods utilized a Waters Acquity ultraperformance liquid chromatograph (UPLC) and a Thermo Scientific Q-Exactive high-resolution/accurate mass spectrometer interfaced with a heated electrospray ionization (HESI-II) source and Orbitrap mass analyzer operated at 35,000 mass resolution. Raw data were extracted, peak identified, and QC processed using Metabolon’s hardware and software. Compounds were identified by comparison to library entries of purified standards or recurrent unknown entities. A more detailed description of the metabolomics methods can be found in Text S1.

10.1128/msphere.00104-22.1 TEXT S1 Additional information on the methods used for the metabolomics analysis and an in-depth description of how we controlled for well-to-well contamination in our error analysis. Download Text S1, DOCX file, 0.02 MB. Copyright © 2022 Breen et al. 2022 Breen et al. https://creativecommons.org/licenses/by/4.0/ This content is distributed under the terms of the Creative Commons Attribution 4.0 International license.

### 16S rRNA gene sequencing.

A subset of samples with sufficient volume available following metabolomics had airway microbiotas also characterized with 16S rRNA gene sequencing. Sputum samples were thawed on ice and then homogenized with 10% Sputolysin (MilliporeSigma, Burlington, MA, USA). DNA extractions for sputum samples and reagent controls were performed with mechanical disruption by bead beating followed by incubation with bacterial lysis buffer (Roche Diagnostics Corp., Indianapolis, IN, USA), lysostaphin (MilliporeSigma, Burlington, MA, USA), and lysozyme (MilliporeSigma, Burlington, MA, USA), followed by treatment with proteinase K (Qiagen, Germantown, MD, USA) as previously described (65). DNA were extracted and purified using a MagNA Pure nucleic acid purification platform (Roche Diagnostics Corp., Indianapolis, IN, USA) according to the manufacturer’s protocol. Prior studies demonstrated stability of bacterial DNA in CF sputum stored at −80°C over a 15-year period (66).

The V4 region of the bacterial 16S rRNA gene was amplified using touchdown PCR with barcoded dual-index primers. The touchdown PCR cycles consisted of 2 min at 95°C, followed by 20 cycles of 95°C for 20 s, 60°C (starting from 60°C, the annealing temperature decreased 0.3°C each cycle) for 15 s and 72°C for 5 min, then followed by another 20 cycles of 95°C for 20 s, 55°C for 15 s, and 72°C for 5 min, and a hold at 72°C for 10 min. The amplicon libraries were then normalized and sequenced on Illumina sequencing platform using a MiSeq reagent kit V2 (Illumina, San Diego, CA, USA). The final load concentration was 4 to 5.5 pM with a 15% PhiX spike to add diversity. Sequencing of the V4 region of the bacterial 16S rRNA gene was performed by the University of Michigan Microbial Systems Molecular Biology Laboratory as previously described (67).

16S sequences were processed with mothur (v.1.43.0) according to the MiSeq standard operating procedure (SOP) (https://mothur.org/wiki/miseq_sop/; accessed March 2020) (68). mothur “shared” and taxonomy files were loaded into phyloseq (1.26.0) in R (3.5.1) for initial processing. Silva v132 (69) was used for the alignment step, and RDP Classifier v16 (70) was used for taxonomic classification. All other mothur settings were set according to the SOP. Samples with fewer than 500 reads and OTUs with average relative abundances less than 0.1% across all samples were removed prior to downstream analyses (2 samples excluded) (71).

### Data analyses. (i) Clinical data.

To examine differences between clinical and demographic variables in the NTM and control sample cohorts, numeric variables were analyzed using Welch’s two-sample *t* test, and categorical and binary variables were analyzed using Fisher’s exact test in base R (3.6.1).

### (ii) Metabolomics data.

For the Metabolon analysis of the complete metabolomics data set, untargeted metabolomics data were processed and analyzed by Metabolon. Specifically, the data were scaled by mass utilized (i.e., the masses of the metabolites in question were used as a scale to establish a high and low end) and then rescaled to set the median to 1. SCFA data were scaled by their root mean square by the base R function “scale” with “center=FALSE.” *q* values were calculated for the complete metabolomics data set (all NTM cases compared to NTM-negative controls) using the methods of Storey and Tibshirani (72). The FDR was calculated as the *q* value that corresponded with the lowest significant *P* value (*P* < 0.05), resulting in a FDR of 20% for the complete metabolomics data set. Missing values were imputed with the minimum value for all metabolites.

### (iii) Microbiome and metabolome data integration.

For the subset of samples that had microbiome data in addition to metabolomics data, the metabolomics data were filtered to remove low variance features using the function nearZeroVar from the caret package (73). Metabolite values were log transformed, and the OTU data set was centered log-ratio (CLR) transformed, taking the log of the ratio between observed frequencies (i.e., data points) and their geometric mean. This was done because the transformation makes the data symmetric and linearly related and places the data in a log-ratio coordinate space (74, 75). The multiblock discriminant analysis was performed using the DIABLO (76) sPLS-DA framework from the mixOmics R package. The design value was set at 0.5 to achieve an even balance between maximizing correlation between omics data sets and maximizing the separation between groups (77). One advantage of sPLS-DA is that it shows the correlations (in the context of this analysis, correlation refers to what is described by the DIABLO group, which is essentially an approximated Pearson correlation calculated from the PLS analysis (78)) between omics data sets that are most relevant for the separation of our groups of interest. Furthermore, sPLS-DA performs a feature selection unlike traditional PLS-DA, resulting in smaller models that are easier to interpret and more generalizable (76). The tune.splsda function was used on each omics data set to determine the optimal number of features and components. In each case, the optimal number of features and components is 1. However, due to the exploratory natures of this study, we chose to set the number of components at 2.

To complement the sPLS-DA results and guide the choice of number of features per component, we used a generalized linear model (GLM) analysis utilizing a cross-validated LASSO approach for feature selection. To generate the GLM, all of the microbial and metabolomic features were entered into to the cv.glmnet function with the following options: α = 1, family = “binomial,” and type.measure = “class.” The model that gave minimum mean cross-validated error was chosen using the s = “lambda.min” option with the coef() function in glmnet. All ordinations, heat maps, and correlation results were generated using the plotIndiv, plotDiablo, cimDiablo, and circoPlot functions of the mixOmics R package with the exception of the of the principal-coordinate analysis (PCoA) and permutational multivariate analysis of variance (PERMANOVA), which were done using the vegan, ape, and phyloseq R packages (76, 79). *q* values for the combined microbiome and metabolome data sets were calculated using the Benjamini-Hochberg method.

### Reproducibility.

Mock community DNA (ZymoBIOMICS microbial community DNA standard) was sequenced to determine sequencing error rates. Water controls were included to assess for PCR contamination, and reagent controls were sequenced to assess for DNA contamination of sputum samples.

### Data availability.

Metabolomics data, mothur log file, OTU tables with taxonomy, and analytic code are available at https://github.com/caverlyl/NTM_metabolomics. Raw sequencing data are available at NCBI (BioProject ID PRJNA594304).
