# Supplementary material for: Sputum Metabolites Associated with Nontuberculous Mycobacterial Infection in Cystic Fibrosis
Source: mSphere. 2022 Apr 28;7(3):e00104-22. doi: 10.1128/msphere.00104-22 (PMC9241540; doi:10.1128/msphere.00104-22)
Supplement: TABLE S1 [file msphere.00104-22-s0002.docx]

|  | NTM positive cases  (N = 17 samples from 15 subjects) | NTM negative controls (N = 26 samples from 26 subjects) | p-value |
| --- | --- | --- | --- |
| Age, years^1^ (Median, IQR) | 20.68 (16.23 ­– 25.2) | 30.85 (23 – 38.15) | < .0001* |
| Sex (% male) ^#^ | 6 (40%) | 15 (58%) | 0.34 |
| CF genotype (no., %)^#^  F508del homozygous  F508del heterozygous  Other | 8 (53%)  7 (47%)  0 | 11 (42%)  10 (39%)  5 (19%) | 0.25 |
| ppFEV_1_ ^1^ | 46 (33 – 76) | 45 (35 - 66) | 0.59 |
| Disease aggressiveness (no., %)  Mild  Moderate  Severe | 3 (18%)  7 (41%)  7 (41%) | 16 (62%)  5 (19%)  5 (19%) | 0.018* |
| Acceptable BMI^1^ | 9 (53%) | 14 (54%) | 1 |
| Current CF respiratory cultures (no., %)^1^  *P. aeruginosa*  MRSA  MSSA  *S. maltophilia*  *Achromobacter* spp.  *Burkholderia* spp.  *Aspergillus* spp. | 7 (41%)  2 (12%)  9 (53%)  2 (12%)  0 (0%)  0 (0%)  4 (24%) | 14 (54%)  4 (15%)  12 (46%)  3 (12%)  1 (4%)  3 (12%)  6 (23%) | 0.54  1  0.76  1  1  0.27  1 |
| CF respiratory cultures, ≥ 1 positive^3^ (no., %)  *P. aeruginosa*  MRSA  MSSA  *S. maltophilia*  *Achromobacter* spp.  *Burkholderia* spp.  *Aspergillus* spp. | 11 (65%)  8 (47%)  12 (71%)  5 (29%)  2 (12%)  1 (6%)  6 (35%) | 19 (73%)  7 (27%)  19 (73%)  5 (19%)  4 (15%)  3 (12%)  13 (50%) | 0.74  0.21  1  0.48  1  1  0.37 |
| Diagnosis of CF-related diabetes^1^ | 6 (35%) | 5 (19%) | 0.30 |
| No. pulmonary exacerbations^2^ (median, IQR) | 3 (1 – 5) | 3 (1 – 5) | 0.96 |
| Chronic azithromycin  Current^1^  History^4^ | 11 (65%)  14 (82%) | 20 (77%)  22 (85%) | 0.49  1 |
| Chronic inhaled antibiotics  Current^1^  History^4^ | 8 (47%)  11 (65%) | 20 (77%)  21 (81%) | 0.057  0.30 |
| Inhaled steroids  Current^1^  History^4^ | 13 (77%)  15 (88%) | 16 (62%)  19 (73%) | 0.34  0.28 |
| CFTR modulators  Current^1^  History^4^ | 4 (24%)  5 (29%) | 7 (27%)  8 (31%) | 1  1 |
| Clinical state^1^  Baseline  Exacerbation  Treatment  Recovery | 7 (41%)  5 (29%)  5 (29%)  0 (0%) | 12 (46%)  8 (31%)  4 (15%)  2 (8%) | 0.88 |

*statistically significant,  **^#^**subject specific comparison
