# Supplementary material for: Sputum Metabolites Associated with Nontuberculous Mycobacterial Infection in Cystic Fibrosis
Source: mSphere. 2022 Apr 28;7(3):e00104-22. doi: 10.1128/msphere.00104-22 (PMC9241540; doi:10.1128/msphere.00104-22)
Supplement: TABLE S3 [file msphere.00104-22-s0004.docx]

| Variable | p-value | coef | q-value |
| --- | --- | --- | --- |
| lactobacillic acid | 7.95E-05 | -1.4172622 | 0.07195685 |
| X - 24585 | 0.00135128 | -0.6937567 | 0.2591099 |
| indolelactate | 0.00138719 | -0.5700027 | 0.2591099 |
| 1-palmitoleoyl-GPC (16:1)* | 0.00163671 | 0.94236573 | 0.2591099 |
| 4-methyl-2-oxopentanoate | 0.00195535 | -0.5978447 | 0.2591099 |
| X - 23654 | 0.00242696 | -0.3681888 | 0.2591099 |
| X - 24408 | 0.00253706 | -0.5745966 | 0.2591099 |
| N-stearoyl-sphingosine (d18:1/18:0)* | 0.00306696 | 0.28815567 | 0.2591099 |
| alpha-ketoglutarate | 0.00326008 | -0.4960614 | 0.2591099 |
| 3-methyl-2-oxobutyrate | 0.00338444 | -0.5117029 | 0.2591099 |
