# Supplementary material for: Sputum Metabolites Associated with Nontuberculous Mycobacterial Infection in Cystic Fibrosis
Source: mSphere. 2022 Apr 28;7(3):e00104-22. doi: 10.1128/msphere.00104-22 (PMC9241540; doi:10.1128/msphere.00104-22)
Supplement: TABLE S4 [file msphere.00104-22-s0005.docx]

|  | Classified as control | Classified as NTM | No consensus |
| --- | --- | --- | --- |
| Control | **21** | **1** | **4** |
| NTM | **2** | **12** | **3** |
