# Supplementary material for: Sputum Metabolites Associated with Nontuberculous Mycobacterial Infection in Cystic Fibrosis
Source: mSphere. 2022 Apr 28;7(3):e00104-22. doi: 10.1128/msphere.00104-22 (PMC9241540; doi:10.1128/msphere.00104-22)
Supplement: TABLE S5 [file msphere.00104-22-s0006.docx]

| Variable | Coefficient |
| --- | --- |
| Veillonella | **0.36740169** |
| X - 23276 | 0.06825589 |
| Haemophilus | 0.02169136 |
| hexanoylcarnitine (C6) | -0.0024163 |
| (R)-3-hydroxybutyrylcarnitine | -0.0224973 |
| palmitoyl-dihomo-linolenoyl-glycerol (16:0/20:3n3 or 6) [2]* | -0.0907301 |
| sphingomyelin (d18:1/20:1, d18:2/20:0)* | -0.1063475 |
| (Intercept) | -1.8776073 |
