# Supplementary figures and images for: Sputum Metabolites Associated with Nontuberculous Mycobacterial Infection in Cystic Fibrosis
Source: mSphere. 2022 Apr 28;7(3):e00104-22. doi: 10.1128/msphere.00104-22 (PMC9241540; doi:10.1128/msphere.00104-22)

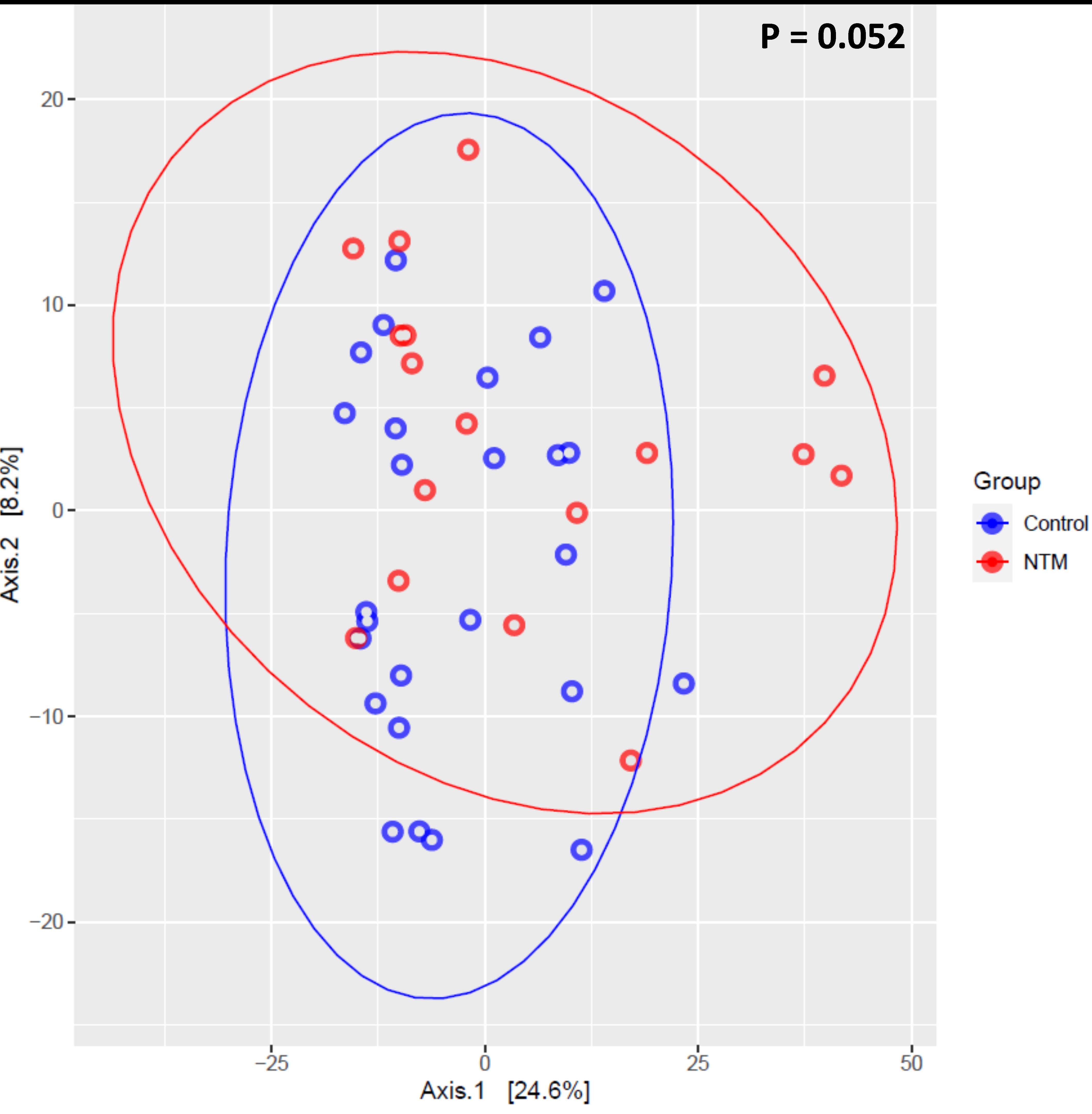

Supplement: FIG S1 [file msphere.00104-22-s0007.tif]

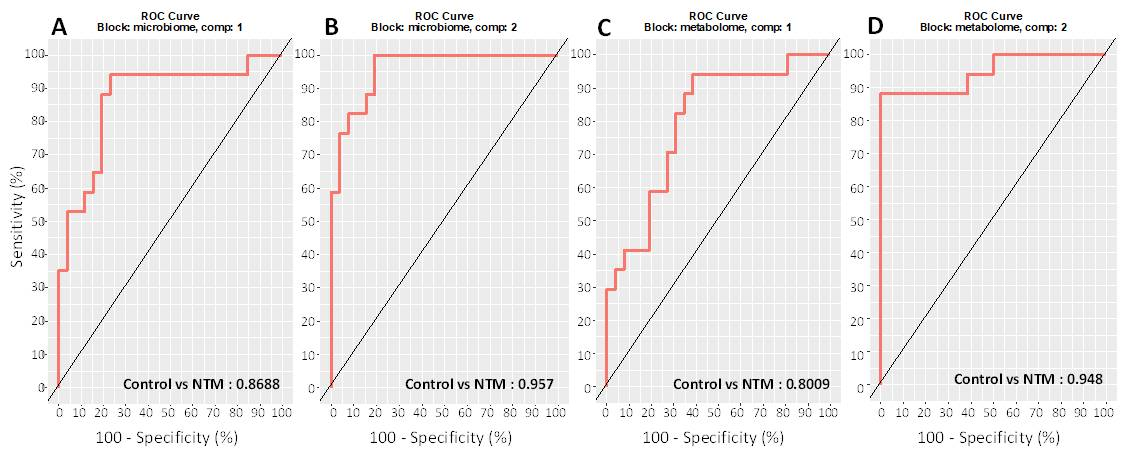

Supplement: FIG S2 [file msphere.00104-22-s0008.tif]

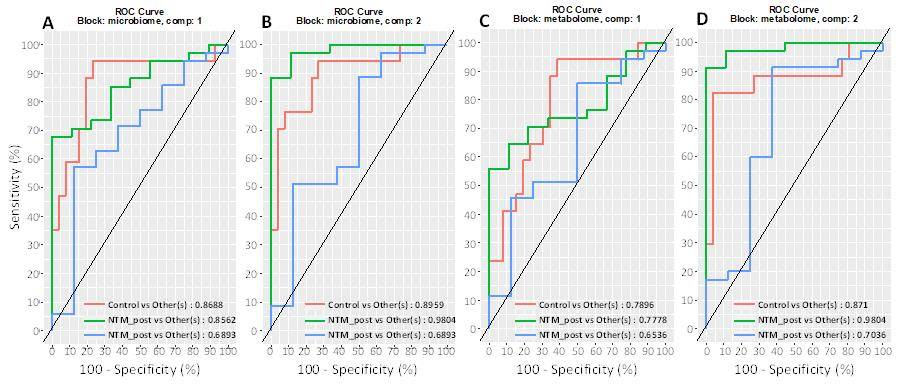

Supplement: FIG S3 [file msphere.00104-22-s0009.tif]

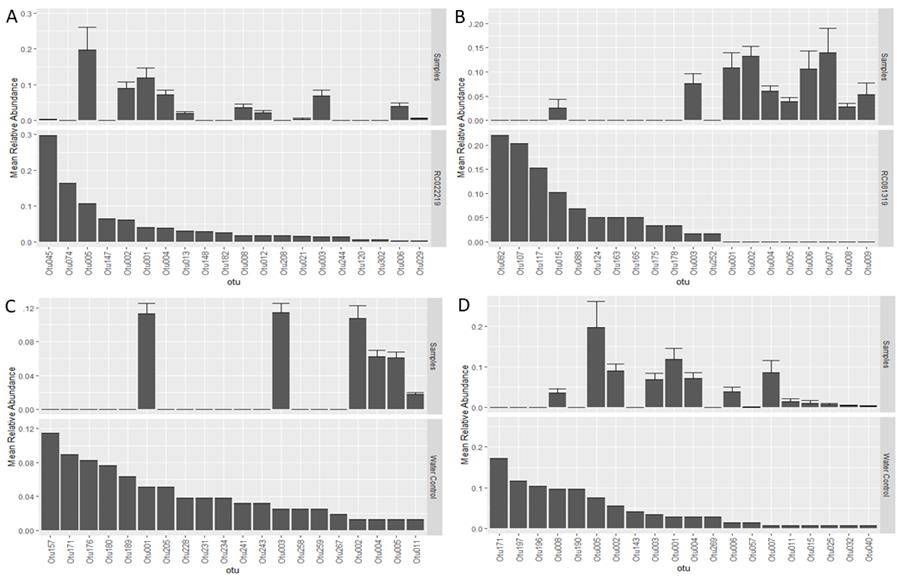

Supplement: FIG S4 [file msphere.00104-22-s0010.tif]
