# Supplementary material for: Sputum Metabolites Associated with Nontuberculous Mycobacterial Infection in Cystic Fibrosis
Source: mSphere. 2022 Apr 28;7(3):e00104-22. doi: 10.1128/msphere.00104-22 (PMC9241540; doi:10.1128/msphere.00104-22)
Supplement: TEXT S1 [file msphere.00104-22-s0001.docx]

**DIFFERENCES IN CF SPUTUM METABOLOMICS BETWEEN PEOPLE WITH AND WITHOUT NONTUBERCULOUS MYCOBACTERIAL INFECTION**

**Supplemental Methods**

Supplemental metabolomics methods:

*Metabolomics*: Sputum samples were shipped on dry ice for short chain fatty acid (SCFA) measurements and untargeted metabolomics (ultrahigh performance liquid chromatography-tandem mass spectroscopy) at Metabolon®, following their standard procedures. Samples were maintained at -80^o^C until processed. For SCFAs analysis, sputum samples were spiked with stable labelled internal standards, homogenized, subjected to protein precipitation with an organic solvent, and then analyzed for eight short chain fatty acids: acetic acid (C2), propionic acid (C3), isobutyric acid (C4), butyric acid (C4), 2-methyl-butyric acid (C5), isovaleric acid (C5), valeric acid (C5) and caproic acid (hexanoic acid, C6) by LC-MS/MS. After centrifugation, an aliquot of the supernatant was derivatized. The reaction mixture was injected onto an Agilent 1290/AB Sciex QTrap 5500 LC MS/MS system equipped with a C18 reversed phase UHPLC column. The mass spectrometer was operated in negative mode using electrospray ionization. The peak area of the individual analyte product ions was measured against the peak area of the product ions of the corresponding internal standards. Quantitation was performed using a weighted linear least squares regression analysis generated from fortified calibration standards prepared immediately prior to each run. LC-MS/MS raw data were collected and processed using AB SCIEX software Analyst 1.6.2.

For untargeted metabolite measurements, samples were prepared using the automated MicroLab STAR® system from Hamilton Company. Recovery standards were added prior to the first step in the extraction process for QC purposes. Proteins were precipitated with methanol under vigorous shaking for 2 min (Glen Mills GenoGrinder 2000) followed by centrifugation. The resulting extract was divided into five fractions: two for analysis by two separate reverse phase (RP)/UPLC-MS/MS methods with positive ion mode electrospray ionization (ESI), one for analysis by RP/UPLC-MS/MS with negative ion mode ESI, one for analysis by HILIC/UPLC-MS/MS with negative ion mode ESI, and one sample was reserved for backup. Samples were placed briefly on a TurboVap® (Zymark) to remove the organic solvent. The sample extracts were stored overnight under nitrogen before preparation for analysis. Several types of controls were analyzed in concert with the experimental samples: a pooled matrix sample generated by taking a small volume of each experimental sample served as a technical replicate throughout the data set; extracted water samples served as process blanks; and a cocktail of QC standards chosen not to interfere with the measurement of endogenous compounds were spiked into every analyzed sample.

All methods utilized a Waters ACQUITY ultra-performance liquid chromatography (UPLC) and a Thermo Scientific Q-Exactive high resolution/accurate mass spectrometer interfaced with a heated electrospray ionization (HESI-II) source and Orbitrap mass analyzer operated at 35,000 mass resolution. The sample extract was dried then reconstituted in solvents compatible with each of the four methods. Each reconstitution solvent contained a series of standards at fixed concentrations to ensure injection and chromatographic consistency. One aliquot was analyzed using acidic positive ion conditions, chromatographically optimized for more hydrophilic compounds. In this method, the extract was gradient eluted from a C18 column (Waters UPLC BEH C18-2.1x100 mm, 1.7 µm) using water and methanol, containing 0.05% perfluoropentanoic acid (PFPA) and 0.1% formic acid (FA). Another aliquot was also analyzed using acidic positive ion conditions; however, it was chromatographically optimized for more hydrophobic compounds. In this method, the extract was gradient eluted from the same afore mentioned C18 column using methanol, acetonitrile, water, 0.05% PFPA and 0.01% FA and was operated at an overall higher organic content. Another aliquot was analyzed using basic negative ion optimized conditions using a separate dedicated C18 column. The basic extracts were gradient eluted from the column using methanol and water, however with 6.5mM Ammonium Bicarbonate at pH 8. The fourth aliquot was analyzed via negative ionization following elution from a HILIC column (Waters UPLC BEH Amide 2.1x150 mm, 1.7 µm) using a gradient consisting of water and acetonitrile with 10mM Ammonium Formate, pH 10.8. The MS analysis alternated between MS and data-dependent MS^n^ scans using dynamic exclusion. The scan range varied slighted between methods but covered 70-1000 m/z. Raw data was extracted, peak-identified and QC processed using Metabolon’s hardware and software. Compounds were identified by comparison to library entries of purified standards or recurrent unknown entities.

Supplemental description of controls for error analysis:

To assess well-to-well contamination, water blanks are sequenced with each plate and run through the mothur MiSeq SOP with associated samples. The 20 most abundant OTUs in the water blank are identified and the abundance of these OTUs is visualized in the associated samples. OTUs found in both the water blanks and sputum are generally the highest abundance OTUs in sputum samples, suggesting some well-to-well contamination. The most abundant OTUs in the water blanks are found in sputum samples only at extremely low levels, if at all, suggesting that while well-to-well contamination is detectable, it is minimal and does not cause significant error in high biomass samples.

Reagent controls are prepared each time a new lot of reagents is opened. The extraction protocol is modified by substituting 350 µL 10% sputolysin for homogenized sputum and proceeding as previously described. After running the reagent control sequences and all associated samples through the mothur MiSeq SOP, potential contamination is assessed by visualizing plots of the relative abundances of the 20 most abundant OTUs in the reagent control and the same OTUs in the experimental samples. Reagent controls show the same pattern as water blanks, with the most abundant OTUs in the reagents generally not detected in samples and OTUs in common generally those in highest abundance in sputum samples. This suggests that the common sequences are the result of minimal well-to-well contamination on the sequencing plate rather than reagent contamination.
